# Matrix-assisted laser desorption ionization time-of-flight mass spectrometry for rapid identification of mold and yeast cultures of *Penicillium marneffei*

**DOI:** 10.1186/s12866-016-0656-0

**Published:** 2016-03-10

**Authors:** Susanna K. P. Lau, Clare S. K. Lam, Antonio H. Y. Ngan, Wang-Ngai Chow, Alan K. L. Wu, Dominic N. C. Tsang, Cindy W. S. Tse, Tak-Lun Que, Bone S. F. Tang, Patrick C. Y. Woo

**Affiliations:** State Key Laboratory of Emerging Infectious Diseases, The University of Hong Kong, Hong Kong, China; Research Centre of Infection and Immunology, The University of Hong Kong, University Pathology Building, Queen Mary Hospital, Hong Kong, China; Carol Yu Centre for Infection, The University of Hong Kong, Hong Kong, China; Department of Microbiology, The University of Hong Kong, Hong Kong, China; Hong Kong Sanatorium Hospital, Hong Kong, China; Department of Pathology, Pamela Youde Nethersole Eastern Hospital, Hong Kong, China; Department of Pathology, Queen Elizabeth Hospital, Hong Kong, China; Department of Pathology, Kwong Wah Hospital, Hong Kong, China; Department of Pathology, Tuen Mun Hospital, Hong Kong, China

**Keywords:** MALDI-TOF, MS, Rapid, Identification, *Pencillium*, *marneffei*

## Abstract

**Background:**

*Penicillium marneffei* is the most important thermal dimorphic fungus causing systemic mycosis in HIV-infected and other immunocompromised patients in Southeast Asia. However, laboratory diagnosis of penicilliosis, which relies on microscopic morphology and mycelial-to-yeast conversion, is time-consuming and expertise-dependent, thus delaying diagnosis and treatment. Although matrix -assisted laser desorption ionization time-of-flight mass spectrometry (MALDI-TOF MS) is useful for identification of various medically important fungi, its performance for identification of *P. marneffei* is less clear.

**Results:**

We evaluated the performance of the Bruker MALDI-TOF MS system for identification of mold and yeast cultures of 59 clinical strains and the type strain of *P. marneffei* using the direct transfer method, with results compared to four phylogenetically closely related species, *P. brevi-compactum*, *P. chrysogenum*, *Talaromyces aurantiacus* and *T. stipitatus*. Using the Bruker original database combined with BDAL v4.0.0.1 and Filamentous Fungi Library 1.0, MALDI-TOF MS failed to identify the 60 *P. marneffei* strains grown in mold and yeast phase (identified as *P. funiculosum* and *P. purpurogenum* with scores <1.7 respectively). However, when the combined database was expanded with inclusion of spectra from 21 *P. marneffei* strains in mold and/or yeast phase, all the remaining 39 *P. marneffei* strains grown in mold or phase were correctly identified to the species level with score >2.0. The MS spectra of *P. marneffei* exhibited significant difference to those of *P. brevi-compactum*, *P. chrysogenum, T. aurantiacus* and *T. stipitatus*. However, MALDI-TOF MS failed to identify these four fungi to the species level using the combined database with or without spectra from *P. marneffei*.

**Conclusions:**

MALDI-TOF MS is useful for rapid identification of both yeast and mold cultures of *P. marneffei* and differentiation from related species. However, accurate identification to the species level requires database expansion using *P. marneffei* strains.

## Background

*Penicillium marneffei* is the most important thermal dimorphic fungus causing respiratory, skin and systemic mycosis in Southeast Asia [1–4]. After its first discovery in bamboo rats [5, 6], only 18 cases of human diseases were reported until 1985 [7]. The HIV pandemic in the 1980’s has resulted in a surge of HIV-associated *P. marneffei* infections in Southeast Asia where the fungus is endemic [2]. In Hong Kong, about 10 % of HIV patients have been infected with *P. marneffei* [8, 9]. In addition to HIV-infected patients, penicilliosis is also an emerging disease in other immunocompromised patients such as transplant recipients and patients on immunosuppressant therapies [10–13]. Imported cases of *P. marneffei* infections have also been reported in non-endemic countries [14, 15]. Despite its medical importance, the mode of transmission, and dimorphic and pathogenic mechanisms of *P. marneffei* remain poorly understood [16–18].

Diagnosis of penicilliosis can be difficult, since many clinical laboratories are inexperienced in identifying this dimorphic fungus. *P. marneffei* exhibits distinct cellular morphologies at different temperatures, in mycelial phase at 25 °C and yeast phase at 37 *°*C [19]. Laboratory diagnosis relies on either direct examination of infected tissues that contain *P. marneffei* yeast-like cells or, more commonly, positive cultures from tissues or blood. Although the production of blue-green to yellowish colonies from cultures at 25 °C with a soluble diffusible red pigment on agar is highly suggestive of *P. marneffei*, other *Penicillium* or related species can also produce similar pigments [20]. Therefore, definitive identification of *P. marneffei* often requires the demonstration of mycelial-to-yeast conversion, which typically shows oval, yeast-like cells with abortive, branched and separate hyphae at 37 °C, and. However, the entire process takes approximately two weeks As a result, the identification of *P. marneffei* is often time-consuming and requires considerable experience, which may lead to delayed diagnosis and treatment.

Conventional phenotypic methods and commercial kits may not readily identify the less commonly encountered dimorphic fungi. On the other hand, sequencing of conserved gene targets for fungal species identification is expensive and requires special expertise. Matrix-assisted laser desorption ionization time-of-flight mass spectrometry (MALDI-TOF MS) has recently emerged as a revolutionary technique for pathogen identification, yielding rapid, accurate and highly reproducible results at a lower price than any other routine methods used in clinical microbiology laboratories, although the equipment itself requires substantial initial investment [21–23]. The methodology is easy to follow even with an inexperienced operator, and the results are available within minutes. Therefore, the technique is now integrated into many clinical laboratories. MALDI-TOF MS is useful for identification of various bacterial pathogens, including those less common species [24–26]. Recent studies have shown that MALDI-TOF MS is also capable of identifying various medically important fungi, including both yeasts, e.g. *Candida* and *Cryptococcus*, and molds, e.g. *Aspergillus*, *Fusarium* and dermatophytes [27–36]. The technique has also been extended to the development of antifungal susceptibility tests for *Candida* and *Aspergillus* [37, 38]. Since only minimal amounts of micro-organisms are required, the technique is also potentially advantageous over conventional identification for “dangerous” pathogens, such as *Burkholderia pseudomallei* and dimorphic fungi, which require biosafety level 3 laboratories for culture [25]. However, there has been no study focusing on the usefulness of MALDI-TOF MS for identification of dimorphic fungi such as *P. marneffei*. In this study, we evaluated the performance of MALDI-TOF MS in identifying both the mold and yeast cultures of 60 *P. marneffei* strains.

## Methods

### Fungal strains and culture conditions

A total of 60 *P. marneffei* strains were included, including 59 clinical strains isolated from patients with culture-documented penicilliosis and *P. marneffei* type strain ATCC 18224^T^ isolates from a bamboo rat [39]. All the 59 *P. marneffei* clinical strains were identified by sequencing of three housekeeping genes, mannose phosphate isomerase (MPI), plasma membrane H+ ATPase (PM-ATPase) and pyruvate kinase (PK), as described previously [40]. The sequences of all three housekeeping genes were identical among the 59 clinical strains and *P. marneffei* type strain ATCC 18224^T^ (data not shown). Since *P. marneffei* is phylogenetically closely related to other *Penicillium* species and *Talaromyces* which is the telemorph of some *Penicillium* species [18, 41], two strains of other *Penicillium* species (*P. brevi-compactum* ATCC 14586 and *P. chrysogenum* ATCC 9480) and two strains of *Talaromyces* species (*Talaromyces aurantiacus* strain PW3105 and *T. stipitatus* ATCC 10500) were also included. *T. aurantiacus* strain PW3105 was isolated from the bronchial trap of a patient with left lung mass. All *P. marneffei* strains were grown on Sabouraud dextrose agar (SDA) (Oxoid, Cambridge, UK) at 37 °C for yeast cultures and at 25 °C for mold cultures for 4 to 7 days as described previously [17, 18]. Yeast and mold forms of *P. marneffei* were collected by scraping and resuspension in 300 μl sterile water. The four strains, *P. brevi-compactum*, *P. chrysogenum*, *T. aurantiacus* and *T. stipitatus* were grown on SDA at 25 °C for 3 to 5 days.

### MALDI-TOF MS

All isolates were tested in duplicates by MALDI-TOF MS using ethanol-formic acid extraction protocol according to the manufacturers’ instructions. Both yeast and mold cultures of *P. marneffei*, and mold cultures of *P. brevi-compactum, P. chrysogenum, T. aurantiacus and T. stipitatus*, were analyzed by the direct transfer method using the same experimental conditions [25, 42]. Briefly, single colonies were washed in 300 μl sterile H_2_O and then resuspended in 900 μl absolute ethanol followed by centrifugation at 13,000 × g for 2 min. The supernatant was then removed and the pellet was allowed to air dry for 5 min. The pellet was mixed with 50 μl 70 % formic acid by vortex. Fifty microliters of 100 % acetonitrile were added to the solution and was mixed by pipetting. After centrifugation at 13,000 × g for 2 min, 1 μl of the supernatant was transferred onto a spot of a polished steel target plate in a thin film (Bruker Daltonik, Bremen, Germany). Each spot was then air-dried and overlaid with 1 μl matrix solution (α-cyano-4-hydroxycinnamic acid in 50 % acetonitrile and 2.5 % trifluoroacetic acid) (Bruker Daltonik). After crystallization of the matrix solution, the target was loaded into the MALDI-TOF MS spectrometer (Bruker Daltonik) for analysis. Spectra were obtained with an accelerating voltage of 20 kV in linear mode and analyzed within an *m/z* charge of 2,000 to 20,000 Da. Spectra were analyzed with MALDI Biotyper™ 3.0 software against the combined database with Reference Library BDAL v4.0.0.1 (Bruker Daltonik) and Filamentous Fungi Library 1.0 (Bruker Daltonik), with or without inclusion of additional spectra from *P. marneffei* strains grown in yeast or mold phase. Since *P. marneffei* is not represented in the Bruker Daltonik database, both yeast and mold cultures of 20 *P. marneffei* strains were later added as reference strains (Table 1). The MALDI Biotyper output is a log (score) in the range 0 to 3.0. Thresholds for species and genus identification were ≥2.0 and ≥1.7 respectively. If the scores from the first run were <2.0, a second run in duplicate was immediately performed. The highest of all the scores was considered the final result. Results are presented with the species or genus identification (above the cutoff scores). Scores below the cutoff were considered invalid results with the conclusion “no identification”. Bruker bacterial test standard (BTS, no. 255343, Bruker Daltonics) was used for calibration and quality control in each run. Obtained spectra were subject to hierarchical cluster analysis using ClinProTools 3.0 (Bruker Daltonics) as described previously [43].

**Table 1:** Comparison of identification results by Bruker database and expanded database with *P. marneffei* strains

Database	Fungi (no. of strains)	Growth form	Top rank identification	Logscore range
Bruker database	*Penicillium marneffei* (60)	Mold	*Penicillium funiculosum*	1.207-1.554
[include BDAL (Bruker, Version 4.0.0.1),	*Penicillium marneffei* (60)	Yeast	*Penicillium purpurogenum*	1.277-1.566
Filamentous Fungi Library 1.0 (Bruker)]	*Penicillium brevi-compactum* (1)	Mold	*Penicillium brevi-compactum*	1.736
	*Penicillium chrysogenum* (1)	Mold	*Penicillium chrysogenum*	1.689
	*Talaromyces aurantiacus* (1)	Mold	*Penicillium purpurogenum*	1.499
	*Talaromyces stipitatus* (1)	Mold	*Pseudomonas putida*	1.343
Expanded Bruker database with inclusion of spectra from 20 *P. marneffei* strains grown in mold form	*Penicillium marneffei* (40)	Mold	*Penicillium marneffei*	2.355-2.631
	*Penicillium marneffei* (40)	Yeast	*Penicillium marneffei*	2.130-2.607
	*Penicillium brevi-compactum* (1)	Mold	*Penicillium brevi-compactum*	1.714
	*Penicillium chrysogenum* (1)	Mold	*Penicillium chrysogenum*	1.689
	*Talaromyces aurantiacus* (1)	Mold	*Penicillium marneffei*	1.604
	*Talaromyces stipitatus* (1)	Mold	*Lactobacillus plantarum*	1.368
Expanded Bruker database with inclusion of spectra from 20 *P. marneffei* strains grown in yeast form	*Penicillium marneffei* (40)	Mold	*Penicillium marneffei*	2.150-2.575
	*Penicillium marneffei* (40)	Yeast	*Penicillium marneffei*	2.264-2.777
Expanded Bruker database with inclusion of spectra from 20 *P. marneffei* strains grown in both mold and yeast form	*Penicillium marneffei* (40)	Mold	*Penicillium marneffei*	2.396-2.628
	*Penicillium marneffei* (40)	Yeast	*Penicillium marneffei*	2.389-2.689

## Results

**Fig. 1:**
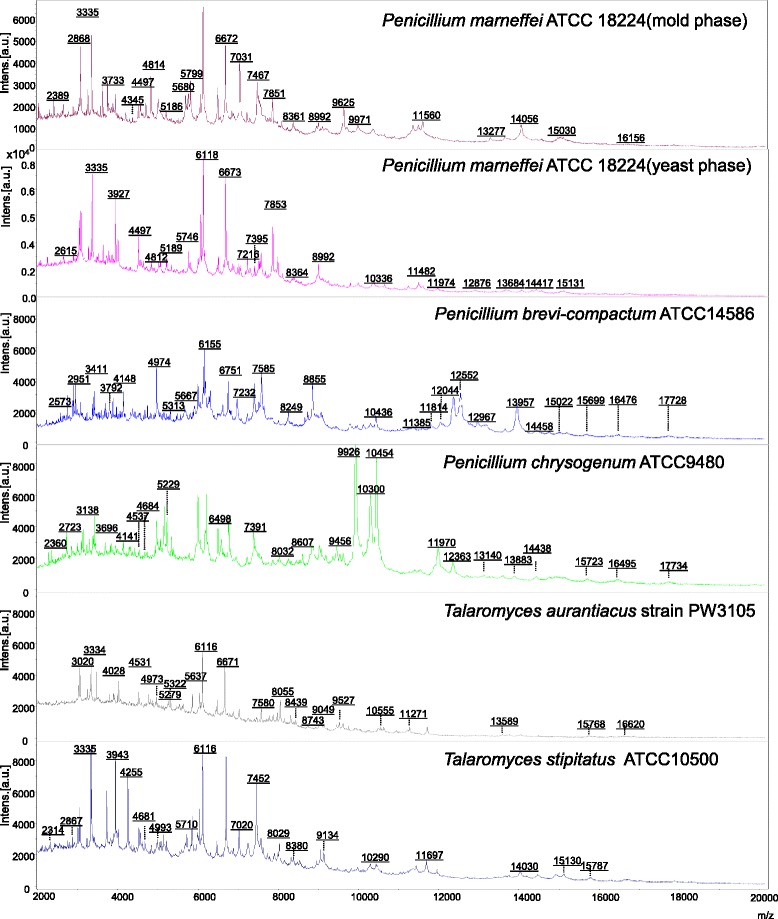
MALDI-TOF mass spectra of mold and yeast cultures of *P. marneffei* compared to those of *P. brevi-compactum*, *P. chrysogenum*, *T. aurantiacus* and *T. stipitatus*.

Representative spectra obtained with mold and yeast cultures of *P. marneffei* compared to those obtained with mold cultures of *P. brevi-compactum*, *P. chrysogenum*, *T. aurantiacus* and *T. stipitatus* are shown in Fig. 1. They exhibited significant difference between the five different fungal species, suggesting that MALDI-TOF MS is potentially useful to differentiate between *P. marneffei* and related fungal species. In contrast, the spectra of the mold and yeast cultures of *P. marneffei* are similar. The MALDI-TOF MS identification results of the 60 *P. marneffei* strains are represented in Table 1. Using the combined database with BDAL v4.0.0.1 (Bruker Daltonik) and Filamentous Fungi Library 1.0 (Bruker Daltonik), without inclusion of additional spectra from *P. marneffei* strains, the 60 *P. marneffei* strains grown in mold phase were identified as *P. funiculosum* with score 1.207-1.554 (indicating no identification); while these 60 strains grown in yeast phase were identified as *P. purpurogenum* with score 1.277-1.566 (indicating no identification). *P. brevi-compactum* and *P. chrysogenum* were correctly identified to the species level but only with score 1.735 and 1.689 respectively (indicating genus level and no identification respectively). *T. aurantiacus* and *T. stipitatus* were identified as *P. purpurogenum* and *Pseudomonas putida* with score 1.499 and 1.343 respectively (indicating no identification).

When the above combined database was expanded with inclusion of spectra from 21 *P. marneffei* strains (including 20 clinical strains and type strain 18224^T^) grown in mold phase, all the remaining 39 *P. marneffei* strains grown in both mold and yeast phase were correctly identified to the species level with score 2.355–2.631 and 2.130–2.607 respectively. *P. brevi-compactum* and *P. chrysogenum* were again identified to the species level but with score 1.714 and 1.689 respectively (indicating genus level and no identification respectively). *T. aurantiacus* and *T. stipitatus* were identified as *P. marneffei* and *Lactobacillus plantarum* with score 1.604 and 1.368 respectively (indicating no identification).

**Fig. 2:**
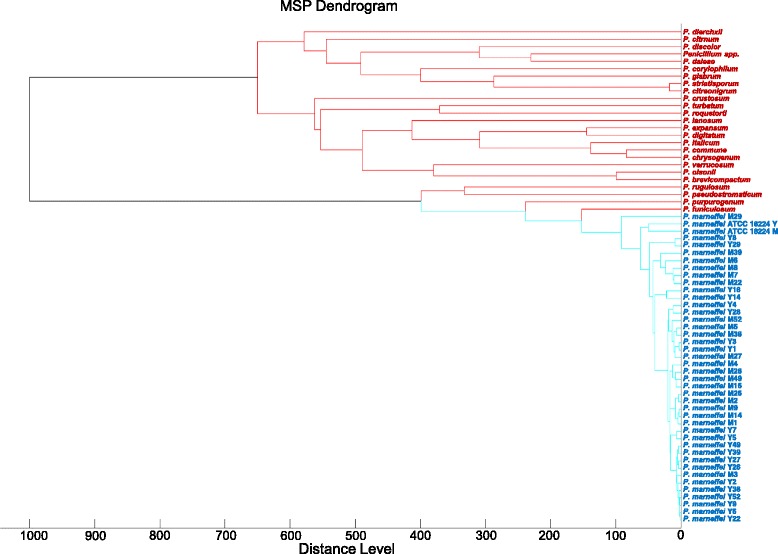
Dendrogram generated from hierarchical cluster analysis of MALDI-TOF mass spectra of mold and yeast cultures of *P. marneffei*. M, mold; Y, yeast.

When the combined database was expanded with inclusion of spectra from the 21 *P. marneffei* strains grown in yeast phase, all the remaining 39 *P. marneffei* strains grown in both mold and yeast phase were also correctly identified to the species level with score >2.0. Similarly, when the combined database was expanded with inclusion of spectra from 21 *P. marneffei* strains grown in both mold and yeast phase, all the remaining 39 *P. marneffei* strains grown in both mold and yeast phase were correctly identified to the species level with score >2.0. Hierarchical cluster analysis showed that the protein mass spectra of mold and yeast forms of *P. marneffei* were clustered together but formed a distinct branch being most closely related to *P. funiculosum* (Table 2, Fig. 2).

**Table 2:** Spectra of *Penicillium* strains used for hierarchical cluster analysis

Species	Strain	Source
*Penicillium dierckxii*	DSM 62842	Bruker database
*Penicillium citrinum*	DSM 1179	Bruker database
*Penicillium discolor*	MPA 1338	Bruker database
*Penicillium sp*	MPA 1326	Bruker database
*Penicillium daleae*	UGB 743	Bruker database
*Penicillium corylophilum*	MPA 11110 01	Bruker database
*Penicillium glabrum*	DSM 16516	Bruker database
*Penicillium striatisporum*	DSM 2439	Bruker database
*Penicillium citreonigrum*	DSM 2427	Bruker database
*Penicillium crustosum*	MPA 1412	Bruker database
*Penicillium turbatum*	DSM 2426 T	Bruker database
*Penicillium roqueforti*	DSM 1079	Bruker database
*Penicillium lanosum*	UGB 411	Bruker database
*Penicillium expansum*	DSM 1282	Bruker database
*Penicillium digitatum*	LLH 16 256 6 4	Bruker database
*Penicillium italicum*	DSM 2417	Bruker database
*Penicillium commune*	MPA 1260	Bruker database
*Penicillium chrysogenum*	MPA 1262	Bruker database
*Penicillium verrucosum*	DSM 12639	Bruker database
*Penicillium olsonii*	DSM 16515	Bruker database
*Penicillium brevicompactum*	DSM 21173	Bruker database
*Penicillium rugulosum*	DSM 19649	Bruker database
*Penicillium pseudostromaticum*	DSM 2421	Bruker database
*Penicillium purpurogenum*	DSM 21170	Bruker database
*Penicillium funiculosum*	MPA 1271	Bruker database
*Penicillium marneffei*	ATCC 18224^T^	Bamboo rat [39]
*Penicillium marneffei*	Mold form (20 strains) Yeast form (20 strains)	Clinical isolates

## Discussion

Despite the usefulness of MALDI-TOF MS for various fungi as demonstrated by many studies [27–35], its performance on dimorphic fungi is less well understood. In one study in which the MALDI-TOF MS database was developed using 294 isolates of various molds, only 10 strains of thermally dimorphic fungi (four *Histoplasma capsulatum* two *Coccidioides immitis*, one each of *Blastomyces dermatitidis*, *Paracoccidioides brasiliensis*, *P. marneffei* and *Sporothrix schenkii*) were included [44]. Although *H. capsulatum* isolates were identified successfully, other dimorphic fungal species including *P. marneffei* were not included for evaluation [44]. In this study, all the 60 *P. marneffei* strains failed to be identified using the combined Bruker and in-house database alone. The best matches were *P. funiculosum* and *P. purpurogenum* for mold and yeast cultures respectively with score <1.7, indicating “no identification”. Nevertheless, fungal isolates, especially blood culture isolates from immunocompromised patients, identified as other *Penicillium* species with low scores using the Bruker Filamentous Fungi Library 1.0, should raise the suspicion of *P. marneffei*. When the combined database is expanded with spectra from 21 *P. marneffei* strains grown in mold, yeast or both phases, all the remaining 39 strains can be correctly identified to the species level with score >2.0. We also showed that different strains of *P. marneffei* displayed very similar MALDI-TOF MS profiles. As a result, no obvious clustering can be observed between the mold and yeast forms of the same strain in the dendrogram (Fig. 2). This suggests that either mold or yeast cultures of *P. marneffei* can be accurately identified by MALDI-TOF MS, which may potentially replace the tedious culture and morphological examination, and allows timely diagnosis and treatment of penicilliosis.

The most critical bottleneck to improve the performance of MALDI-TOF MS for identifying the less commonly encountered pathogens mainly lies in the composition of the database. Despite the scarcity of reports on the used of MALDI-TOF MS for identifying dimorphic fungi, a recent study tested various clinically encountered molds including *P. marneffei* [45]. The MALDI-TOF Bruker Biotyper system was unable to identify all 28 *P. marneffei* isolates*.* However, using a newly created database with one *P. marneffei* strain, 85.7 % of their *P. marneffei* strains can be accurately identified with score ≥2.0. This is in line with the present results, and suggests that adding more spectra for *P. marneffei* should further improve the success rate of identification as shown in this study. In this respect, we are also willing to share our *P. marneffei* MALDI-TOF MS spectra with interested laboratories to assist the early diagnosis of penicilliosis. The suboptimal database is also likely the reason for the failure in identifying the two other *Penicillium* and two *Talaromyces* species. Although *P. brevi-compactum* and *P. chrysogenum* can be correctly identified to their respective species as the best matches using databases with or without spectra from *P. marneffei*, the scores were below the cutoff (2.0) required for species identification. According to the interpretative criteria for Bruker database, the present *P. brevi-compactum* strain would be identified to the genus level as *Penicilllium* species (scores 1.714 to 1.736) and the *P. chrysogenum* strain would be unidentified (scores 1.689). The low scores may be explained by the limited number of spectra for the two species (five spectra for each species) in the Bruker Filamentous Fungi Library, which is inadequate to accommodate intraspecies variability. On the other hand, *T. aurantiacus* and *T. stipitatus* would be unidentified according to the same criteria (scores for best matches 1.343-1.604). While *T. aurantiacus* showed the best match to *P. purpurogenum* or *P. marneffei*, surprisingly, *T. stipitatus* showed the best match to the distantly related bacterial species, *P. putida* or *L. plantarum*. This also reflects the lack of spectra for the genus *Talaromyces* and related spectra in the database. *Penicillium* and *Talaromyces* species other than *P. marneffei* are occasionally cultured from clinical specimens such as infected nails. Therefore, inclusion of more spectra from each of these species may improve the performance of MALDI-TOF MS for their identification, and help better understand their disease associations.

Although MALDI-TOF MS is proven to be useful for identification of various bacterial and fungal pathogens, commercial databases may not contain the less commonly encountered pathogens, newly discovered species or those pathogens limited to certain geographical areas. We have previously shown that MALDI-TOF MS is useful for identifying various “difficult-to-identify” bacterial pathogens, as well as *Burkholderia pseudomallei* which is also endemic in southeast Asia [24, 25]. However, the accurate identification of these less common pathogens often requires the expansion of exsiting databases using bacterial strains from the respective species. Since commonly encountered pathogens are often readily identified by phenotypic tests, it is anticipated that the major role of MALDI-TOF in clinical laboratories would be for the identification of the less common or “difficult-to-identify” pathogens. Therefore, further improvement of commercial databases is important to allow the use of MALDI-TOF MS for routine fungal identification. Genotypic methods, such as sequencing of internal transcribed spacer regions, still remain as the current gold standard for fungal identification, which can be partly explained by vast sequence data available in public databases such as GenBank. However, sequencing is still expensive and time-consuming to be routinely used in clinical laboratories. With improvement of commercial databases, MALDI-TOF MS may replace genotypic methods as a cost-saving, first-line identification system for the less common fungal pathogens*.* Compared to other MALDI-TOF MS systems such as Vitek MS which also used for identification of various bacteria and fungi, the Bruker system has the advantage of being more versatile and allowing database enlargements [46]. Therefore, the Bruker system should be considered by laboratories which foresee the need of expanding databases for identifying less common pathogens such as *P. marneffei*.

## Conclusions

MALDI-TOF MS is useful for rapid identification of both yeast and mold cultures of *P. marneffei*. However, accurate identification to the species level requires expansion of the database using *P. marneffei* strains. In countries where *P. marneffei* is endemic, expansion of MALDI-TOF MS databases using different *P. marneffei* strains should be considered in clinical microbiology laboratories. This can be easily achieved using yeast cultures which are safer to handle than mold culture, with only tiny amount of fungal cells from a single colony subject to ethanol-formic acid extraction. The use of this low-cost, rapid and easy-to-perform state-of-the-art technology may help expedite laboratory diagnosis and treatment of penicilliosis.

## Ethics approval and consent to participate

Not applicable

## Consent for publication

Not applicable

## Availability of data and material

We are willing to share the MALDI-TOF MS spectra of our *P. marneffei* strains with interested researchers and laboratories.

## Abbreviations

ATCC: American type culture collection
HIV: human immunodeficiency virus
MALDI-TOF: Matrix-assisted laser desorption ionization time-of-flight mass spectrometry
